# MAPPING THE COSTS AND SOCIOECONOMIC CHARACTERISTICS INVOLVED IN TRAUMATIC BRAIN INJURIES: A SCOPING REVIEW

**DOI:** 10.2340/jrm.v56.18311

**Published:** 2024-08-05

**Authors:** Fanny CROZES, Cyrille DELPIERRE, Nadège COSTA

**Affiliations:** 1 Health Economic Unit, University Hospital Center of Toulouse, Toulouse; 2 EQUITY Research Team, Center for Epidemiology & Research in POPulation Health (CERPOP), UMR 1295, University Toulouse III Paul Sabatier, Toulouse; 3 Institute of Nursing Training, Toulouse University Hospital, Toulouse, France

**Keywords:** health economics, nurse, scoping, socioeconomic characteristics, TBI

## Abstract

**Objective:**

To identify the articles in the existing literature that analyse healthcare costs according to the socioeconomic position (pre- or post-injury) for traumatic brain injury survivors. Secondary aims were to describe the types of costs and socioeconomic characteristics and to determine whether socioeconomic characteristics affect the risk of traumatic brain injury or whether the consequences of trauma alter living conditions post-injury.

**Methods:**

This scoping review followed the methods proposed by Arksey and O’Malley. The literature search was performed in 5 databases.

**Results:**

Twenty-two articles were included, published between 1988 and 2023. Only 2 articles (9%) followed the guidelines for economic evaluation of healthcare programmes and 2 articles (9%) evaluated socioeconomic position “completely” with 3 main individual measures of socioeconomic characteristics (i.e., education, income, and occupation). The relationship between costs and socioeconomic characteristics could vary in 2 ways in traumatic brain injury: socioeconomic disadvantage was mostly associated with higher healthcare costs, and the cost of healthcare reduced the survivors’ living conditions.

**Conclusion:**

This work highlights the need for a detailed and methodologically sound assessment of the relationship between socioeconomic characteristics and the costs associated with trauma. Modelling the care pathways of traumatic brain injury would make it possible to identify populations at risk of poor recovery or deterioration following a TBI, and to develop specific care pathways. The aim is to build more appropriate, effective, and equitable care programmes.

## LAY ABSTRACT

Victims of traumatic brain injuries often need care or help at the time of the trauma but also several months or years after the trauma. Here we are interested in the economic burden of trauma. We know that a disadvantaged person (low income, low level of education for example) is more likely to have a trauma and more complications. However, we do not know if deprivation changes the costs and trajectories of care and if delivered care is effective in the same way for everyone. This scoping review found that few studies measure costs according to people’s socioeconomic position. Available studies showed that the costs seem higher for a disadvantaged person, but also that the living conditions would be lower after the trauma (i.e., reduced social role or level of income or type of employment). There is therefore a need to carry out well-conducted research to correctly measure the costs according to these characteristics and to offer care adapted to individuals.

The 2016 Global Burden of Disease Study (1) suggests that the overall incidence rate of traumatic brain injuries (TBI) has been estimated at 369 cases per 100,000 people per year, which represents approximately 27 million people affected. Other studies estimate the global incidence of TBI worldwide at between 73 and 939 cases per 100,000 people each year (2). This wide range of incidence estimation is partly due to income levels and prevention policies in the various countries, as well as to different data collection methodology (3, 4). TBI is a major cause of death, with 31% occurring during the initial hospital phase (5). This mortality persists with a rate of 27% 1 year after the trauma (6). Sequelae also lead to a 64% persistence of moderate disability at 1 year, difficulties in returning to work, and an altered quality of life (7), despite progress in rehabilitation (8).

Regarding the determinants of TBI, socioeconomic characteristics (in pre-injury or post-injury) have been shown to be associated with trauma or its consequences. The level of income, the level of education, or even isolation are linked to the occurrence of accidental acute events, such as falls and TBI (9–11). Disability related to TBI seems to be greater in patients who live in rural areas, which may be explained by difficult access to rehabilitation care (12). The literature highlights that TBI affects men and people under the age of 40 most commonly and severely; additionally, those with a traumatic head injury and a lower education level experience poorer health than those with a higher level of education (9, 13, 14).

TBI, and its severity, has significant medical and social consequences, and also constitutes an economic burden to the whole of society. Leibson et al. measured this extra cost to society up to 6 years after the injury by comparing people with brain injuries with a control population (15). The average extra cost of a TBI was US$22,000 during the acute phase and the extra cost persisted up to 6 years after the injury. The overall cost for all brain conditions is comparable to the sum of the costs associated with cardiovascular disease, cancer, and diabetes combined (16).

The consideration of costs according to social inequalities in health is essential, to measure their extent and also to estimate the cost burden on health systems attributable to social inequalities in order to rethink the societal model and deliver equitable care (17). Moreover, the view of health professionals is more in favour of equity or efficiency, depending on the country (18, 19). Decision-making of policy-makers needs to answer these questions concerning the optimal allocation of limited resources (20). Recent work has evaluated the relationship between the cost of care in the community or emergency department care, and social inequalities in health, particularly in European countries where the cost of care is a collective responsibility (21–24). Two main hypotheses can be made from the literature, i.e., (*i*) that social disadvantage accentuates comorbidities and makes care paths more complex and costlier, and (*ii*) that most disadvantaged people are at a greater risk of poor recovery and are therefore more likely to require more expensive care. But the literature considering both costs and social determinants is sparse, and so we still do not know whether geographical and socioeconomic characteristics modify the efficiency of the healthcare system. The questions of efficiency and equity therefore remain unanswered. However, the consideration of costs according to social inequalities in healthcare is essential, to measure their extent and also to estimate the cost burden on healthcare systems attributable to social inequalities in order to rethink the societal model and deliver equitable care (17).

Thus, the goal of this scoping review is to identify articles in the literature that analyse the healthcare costs for TBI survivors according to socioeconomic position, and summarize methods and results.

## METHODS

According to Arksey and O’Malley (25), Anderson et al. (26), and Levac et al. (27), a scoping review can have four main purposes: (*i*) to examine the extent, range, and nature of -research activity; (*ii*) to determine the value of undertaking a full systematic review; (*iii*) to summarize and disseminate research findings; and (*iv*) to identify research gaps in the existing literature. Scoping reviews follow a rigorous methodology and allow an initial assessment of what has been done on the basis of heterogeneous data that do not allow a precise question to be answered like the systematic review. This work follows the specific PRISMA recommendations for scoping reviews (PRISMA-ScR).

### Stage 1: Identification of the research question

Our research question was to identify the articles in the existing literature that analyse healthcare costs according to the socioeconomic position (pre-injury or post-injury) for TBI survivors. Then, 2 secondary objectives were pursued: (*i*) to describe the types of costs and socioeconomic characteristics present in the literature, and (*ii*) to determine whether socioeconomic characteristics affect the risk of TBI or whether the consequences of TBI alter the living conditions post-TBI.

### Stage 2: Identifying relevant studies

In collaboration with a research librarian, we targeted different relevant databases and developed a search strategy using free-text terms for databases without thesauri and medical subject headings (MeSH) for databases with thesauri. Databases were selected based on the subject of this research. The literature search was performed in 5 different electronic databases to gather relevant literature: Medline, EMBase, Cochrane, International HTA database, and Web of Science. Note that the databases EMBase and International HTA database did not retrieve any new references.

All publications dated up to January 4, 2023 were included, with no limitations regarding the publication dates. The search terms are listed in Table SI. The type of publication was not filtered as recommended by O’Brien et al. (28). To be eligible for inclusion in this scoping review, articles were required to analyse healthcare costs according to the socioeconomic position of TBI survivors. Articles that assessed costs but not socioeconomic characteristics were excluded. Articles that assessed socioeconomic characteristics but not costs were excluded. Articles on injuries not specified as a head injury were excluded, as were those written in languages other than English or French. Lastly, books were excluded.

### Stage 3: Selection of studies

The first researcher made an initial selection from the results obtained in the different databases. A first screening was done on the basis of the titles, then a second on the basis of the abstracts of the selected articles, to check that the articles used data related to costs and socioeconomic position. The full texts of the selected articles were then reviewed by 2 independent researchers. The exclusion of each article was based on a consensus of the 2 researchers. For the selected articles, the quality assessment (QualSyst tool (29)) of the articles was also done in a standardized way between the 2 researchers to reach consensus.

### Stage 4: Charting data with critical appraisal

The data were extracted using a standardized form for each relevant article: first author, year of publication, study location, study design, patients’ characteristics, study sample, aims of the study, costs measures, socioeconomic characteristics measures, and the quality of the study. Socioeconomic characteristics and costs were described as presented in the articles and details of these variables are also specified in Table SII.

In order to deal with the heterogeneity of the articles and to propose elements of comparison, the economic variables were described and homogenized according to guidelines (30, 31). Direct costs are the resources used in the management of the injury or its adverse effects, including direct medical costs, directly related to the injury, as well as direct non-medical costs, related to the consequences of the management of the injury. Indirect costs are those costs that are indirectly related to the injury and have an impact on productivity. The intangible costs concern the consequences of the injury in terms of reduced well-being for the patient and family. If the perspective assumed is that of the payer, only direct costs are included. A patient perspective considers only costs that are relevant to patients, such as out-of-pocket or intangible costs. The perspective of the hospital considers costs attributable to institutions, such as equipment costs. The societal perspective includes all health effects and costs, including production losses due to illness. This is the most comprehensive perspective.

In the literature, 3 main individual measures are found to assess socioeconomic position, including income level, occupation or socio-professional category, and education level (32–35). These data are rarely collected routinely and are absent from healthcare databases. Therefore, an approximation of these individual data can be made on the basis of ecological indicators. Socioeconomic characteristics were identified and specified for each article. These characteristics have been collected and labelled for each article to enable comparison. Then, the type of measure was specified, such as individual characteristics and ecological indices. Individuals’ characteristics included education level, socio-professional category, income level, poverty rate, marital or living situation, race, or geographic region. The ecological indices use residential area variables to obtain an ecological index, such as household income by zip code or more specific indices related to deprivation level. Lastly, the assessment of socioeconomic individual characteristics was categorized as “partial” or “complete” based on the evaluation of 3 dimensions: education level, income, and socio-professional category. Also, an approximation of socioeconomic position can be made on the basis of ecological deprivation indices (36). The evaluation of ecological indicators was identified for each article (yes/no).

In accordance with guidelines for scoping reviews, the quality assessment of evidence can be performed where it assists with achieving the aim of the scoping review (28, 37). A data quality assessment method was then used, based on the critical appraisal tool QualSyst from Kmet et al. (29), which allows quantitative and qualitative articles to be systematically evaluated and averages of evaluation to be obtained, which then allow the quality of the sources to be subdivided into 4 categories: strong (score > 0.8), good (score between 0.79 and 0.6), adequate (score between 0.59 and 0.5), or poor (score < 0.5).

### Stage 5: Collation, synthesis, and reporting results

The analysis of the meaning of the articles made it possible to position socioeconomic characteristics and costs as related to TBI or consequences of TBI. The articles acknowledge the temporality of events. A classification according to the temporality of the socioeconomic characteristics and costs in relation to the TBI has thus been done. It is important to specify that this is not a causal inference process but rather a description of the temporality of events based on literature data and their classification as pre-existing causal relationships or outcomes (see more about incorrect causal interpretation from Haber et al. (38)).

## RESULTS

**Fig. 1:**
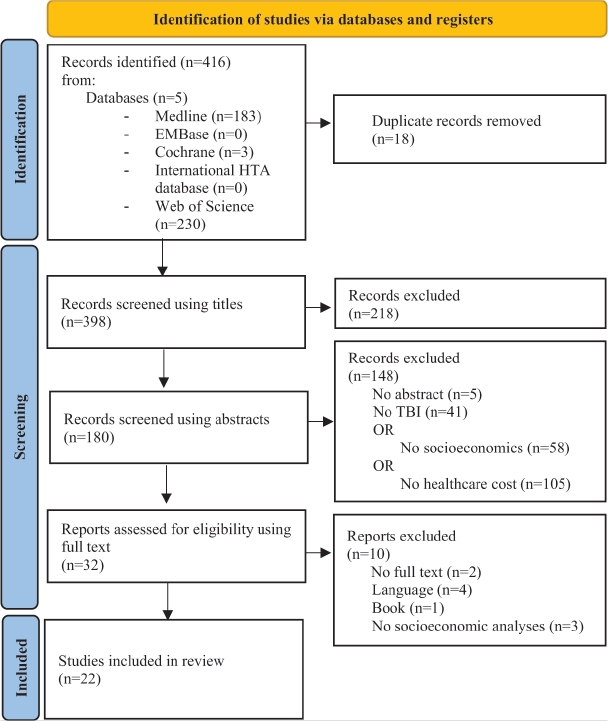
Flow diagram of the selection process according to PRISMA-ScR.

Our search produced 416 records. After the removal of duplicated records, 398 records were screened. A first screening on titles resulted in 180 records, with a second screening on abstracts leaving a total of 32 articles for assessment. Ten more articles were then excluded. Ultimately, 22 articles were included in this scoping review. A final update was made in January 2023 (a flowchart of the selection process according to PRISMA-ScR is shown in Fig. 1 (39–60)).

### Descriptive presentation of results

The descriptive results are presented in Table I. All the articles were published between 1988 and 2022. However, the topic was not very well explored in the period 1988–1999 (just a single article), and there was then an increasing trend in this research topic with 8 articles in 2000–2011 (36%) and 13 articles in 2012–2022 (59%). The majority of the studies were conducted in the USA (*n* = 17, 77%), Australia (*n* = 2, 9%), and Europe (*n* = 2, 9%). Most of the articles were quantitative studies (*n* = 20, 90%), with 14 (64%) adopting retrospective designs, using data from patient medical records and various databases (national registries or healthcare databases). Seven studies were prospective (32%), including 2 cross-sectional studies (9%), 1 qualitative exploratory survey (5%), and 1 literature review (5%). In 4 articles (18%), the objectives are general and do not mention economic or socioeconomic terms (48, 49, 54, 57). Regarding the methodological quality of the studies, 2 studies (9%) were rated as “adequate”, 9 as “good” (41%), and 11 as “strong” (50%).

**Table I:** Description of selected articles

First author, year of publication	Study location	Design of study	Study sample	Objectives	Quality of study
Worthington, 2006	UK	Multi-center prospective cohort study	133 TBI	To update a cost-outcome evaluation, based on a new cohort of adults with severe brain injury, in response to an expansion of neurobehavioral rehabilitation services across the UK	Adequate
Boop, 2016	USA	Retrospective cohort study	213 TBI	To characterize the abusive head trauma population in the geographic area served by Le Bonheur Children’s Hospital and calculate incidence rates and costs of hospital charges	Good
Spitz, 2016	Australia	Retrospective cohort study	798 TBI	To develop competing, predictive multivariate models of costs accrued from the time of the initial accident and over the subsequent 10 years following injury	Strong
Dengler, 2020	USA	Retrospective cohort study	1447 TBI	To determine what the rates of secondary overtriage were in patients with complicated mild traumatic brain injury and to study how they may affect the allocation of healthcare resources	Strong
Norup, 2020	Denmark	Retrospective case-control study	18,328 TBI 89,155 controls 25,708 TBI family members 135,325 control family members	To investigate if a TBI population had increased (*i*) utilization of healthcare costs for the patient and the closest relatives, (*ii*) risk of job loss for the patient and the closest relatives, and (*iii*) risk of divorce, compared with a matched healthy control group	Strong
Tilford, 2005	USA	Retrospective cohort study	98,023 TBI	To examine the incidence, utilization of intracranial pressure monitoring, and outcomes for critically ill children hospitalized with traumatic brain injury	Strong
Zonfrillo, 2016	USA	Retrospective cohort study	1061 TBI	To examine the association between zip code-level median annual household income and costs of hospitalization among severely injured children	Strong
Shafi, 2019	Australia	Retrospective cohort study	3129 TBI	To characterize the sample of workers who had sustained a work-related mild TBI and to assess the influence of assault, as a mechanism of injury, on time away from work	Strong
Kelly, 2022	USA	Retrospective cohort study	19,848 TBI	To evaluate socioeconomic and health disparities among children hospitalized after a TBI	Strong
Johnstone, 2003	USA	Prospective longitudinal study	35 TBI	To characterize financial and vocational outcomes among persons with TBI in terms of employment status, earned and private income, and public assistance received at the time of injury and at 1 year after the injury	Good
McMordie, 1988	USA	Cross-sectional survey	28 TBI, 100 parents, 50 spouses, and 11 siblings	To explore various parameters of the financial costs of head injury for the family	Good
Shigaki, 2009	USA	Prospective longitudinal study	49 TBI	To examine the long-term financial and vocational outcomes for TBI at 2 years post-injury	Good
Ramey, 2019	USA	Retrospective case review	64 TBI	To examine patterns of neurotrauma and associated healthcare utilization in patients treated as a result of unauthorized border crossings by jumping over the USA–Mexico border wall	Adequate
Piatt, 2012	USA	Retrospective cohort study	14,932 TBI	To document the impact of changes during the end of the last century and the beginning of the current one (automotive safety engineering, car seats, bicycle helmets, development of tertiary children’s medical centres, protocol-driven management in the healthcare facilities, etc.) to the practice of neurosurgery on children with a severe TBI	Good
Hart, 2005	USA	Prospective longitudinal study with retrospective self-assessment	94 TBI	To investigate the contribution of pre-injury differences and potential biases in outcome measurement in explaining outcome differences between White and Black persons with moderate and severe TBI	Good
Klevens, 2017	USA	Retrospective case-control study	380 TBI	To examine whether states’ earned income tax credits (EITC) are associated with state rates of hospital admissions for abusive head trauma among children under the age of 2 among states with and without a state EITC	Good
Schneier, 2006	USA	Retrospective cohort study	25,783 TBI	To examine the influence of sociodemographic characteristics and healthcare system factors on the utilization of hospital resources by US children aged 17 and under with a diagnosis of traumatic brain injury	Strong
Relyea-Chew, 2009	USA	Cross-sectional survey	186 (93 TBI or SCI who filed for bankruptcy + 93 controls)	To estimate the prevalence of bankruptcy with substantial medical debt, comparing bankruptcy petitioners with TBI and spinal cord injury with a cohort of randomly selected bankruptcy petitioners	Strong
Yue, 2020	International	Literature review	18 articles (TBI and contusions)	To provide a comprehensive review of the current evidence on rural mild TBI/concussion epidemiology, risk factors, management, and prevention efforts in rural settings	Good
Reynolds, 2001	USA	Exploratory survey	42 state administrators and 12 trauma centre social workers	To identify and describe the barriers faced by patients with a new TBI and evaluate to what extent state Medicaid programmes fund post-acute rehabilitation services	Good
Graves, 2019	USA	Retrospective cohort study	387,846 TBI	To compare healthcare costs and service utilization associated with mild TBI in rural and urban commercially insured children	Strong
Salik, 2022	USA	Retrospective cohort study	26,417 TBI	To analyse the demographic characteristics, TBI severity, insurance status, procedural interventions, complications, and clinical outcomes of paediatric patients who presented with TBI using propensity score matching	Strong

EITC: earned income tax credits; TBI: traumatic brain injury; UK: United Kingdom; USA: United States of America.

### Thematic organization of results

The labelling of data concerning costs and socioeconomic characteristics is presented in Table II, enabling the different variables used to be identified, harmonized, and compared with the studies. With reference to the cost typologies, 4 types of costs were found: direct medical costs, direct non-medical costs, indirect costs, and intangible costs. One typology defined as “other” concerned government assistance (States’ earned income tax credits, Federal income supplement programme or Government financial assistance established before or after TBI). Only 2 studies presented their perspective as recommended in guidelines for reporting cost analyses (9%). In the absence of this specification, the perspectives could be guessed, depending on the types of costs and orientations of the studies. The patient perspective was determined for 4 articles (18%), only 1 of which measured intangible costs (5%), the payer perspective for 8 articles (36%), and the societal perspective – the most complete perspective – for 10 studies (46%).

**Table II:** Typology of costs and socioeconomic characteristics

First author, year of publication	Costs			Socioeconomic characteristics		
	Cost outcomes used in study	Perspective of cost	Type of costs	Socioeconomic characteristics used in the study (other than gender and age)	Type of socioeconomic measures	Individual socioeconomic measures**
Norup, 2020	Hospital costs Medical costs Prescribed medication Job loss	Societal perspectives*	Direct medical costs and indirect costs	Marital status or living situation Income level Race	Individual measures	Partial
Worthington, 2006	Care prior to and after rehabilitation (with hospital or therapy charges and informal support) Rehabilitation costs	Societal perspectives*	Direct medical costs and indirect costs	Geographic region Socio-professional category	Individual measures	Partial
Boop, 2016	Hospital charges (without professional fees) Insurance status	Healthcare payer perspective	Direct medical and non-medical costs	Race Geographic region	Individual measures	Partial
Spitz, 2016	Hospital costs Medical costs Paramedical costs Long-term care costs (with support school and home `accommodation)	Societal perspectives*	Direct medical and non-medical costs and indirect costs	Education level Marital status or living situation Geographic region Socio-professional category	Individual measures	Partial
Dengler, 2020	Transportation charges Hospitalization charges Total charges Insurance status	Healthcare payer perspective*	Direct medical and non-medical costs	Race	Individual measures	Partial
Tilford, 2005	Acute care hospitalization costs Total costs Total charges Insurance status	Healthcare payer perspective*	Direct medical and non-medical costs	Household income by zip code Race Geographic region	Individual and ecological measures	Partial
Zonfrillo, 2016	Hospital costs Payer	Societal perspective*	Direct medical and non-medical costs, and indirect costs	Race Household income by zip code	Individual and ecological measures	Partial
Kelly, 2022	Cost of the hospital stay	Healthcare payer perspective*	Direct medical costs	Race Household income by zip code Geographic region	Individual and ecological measures	Partial
Johnstone, 2003	Government financial assistance	Societal perspective	Others (governmental investment fund for improvements in access to healthcare services)	Socio-professional category Income level	Individual measures	Partial
Shigaki, 2009	Public assistance	Societal perspective*	Others (governmental investment fund for improvements in access to healthcare services)	Race Marital status or living situation Education level Geographic region Socio-professional category Income level	Individual measures	Complete
Ramey, 2019	Physician total charges Inpatient hospital charges	Healthcare payer perspective*	Direct medical costs	Race	Individual measures	Partial
Piatt, 2012	Hospital charges Primary expected payer	Healthcare payer perspective*	Direct medical and non-medical costs	Race Poverty rate Household income by zip code	Individual and ecological measures	Partial
Klevens, 2017	Earned income tax credits (EITC)	Societal perspective*	Others (governmental investment fund for improvements in access to healthcare services)	Race Education level Socio-professional category Poverty rate	Individual measures	Complete
Schneier, 2006	Total charges (do not include professional fees and charges that are not covered) Primary expected payer	Healthcare payer perspective*	Direct medical and non-medical costs	Race	Individual measures	Partial
Yue, 2020	Insurance status Healthcare costs	Societal perspective*	Direct medical and non-medical costs, and indirect costs	Geographic region Socio-professional category	Individual measures	Partial
Reynolds, 2001	If a home and community-based waiver for these patients is available If a Medicaid waiver programme that would cover post-acute care is available If alternative programmes/funding streams are available	Societal perspective*	Direct medical and non-medical costs, and indirect costs	Poverty rate	Individual measures	Partial
Graves, 2019	Total healthcare costs Costs for physical therapy or occupational therapy Costs for speech therapy Costs for psychiatry/psychology treatment	Healthcare payer perspective*	Direct medical costs	Geographic region	Individual measures	Partial
Shafi, 2019	Time away from work	Patient perspective*	Indirect costs	Socioeconomic status (from IRSAD index) Socio-professional category	Individual and ecological measures	Partial
McMordie, 1988	Doctors and hospital bills Medications and others medical expenses Legal expenses Structural modifications in home Specialized therapy	Patient perspective*	Direct medical and non-medical costs	Income level	Individual measures	Partial
Hart, 2005	Home integration Social integration Productivity	Patient perspective*	Intangible costs	Race Education level Marital status or living situation	Individual measures	Partial
Relyea-Chew, 2009	Medical care sponsor Secured debts (e.g., a mortgage) Unsecured debts (priority debt (e.g., taxes, or alimony), nonpriority debts (e.g., credit card), and debts owed to medical or dental service providers) Medical and dental expenses	Patient perspective*	Direct medical and non-medical costs, and indirect costs	Race Household income by zip code Marital status or living situation	Individual and ecological measures	Partial
Salik, 2022	Hospital charges	Healthcare payer perspective*	Direct medical costs	Household income by zip code Poverty rate	Individual and ecological measures	Partial

EITC: earned income tax credits; IRSAD: Index of Relative Socioeconomic Advantage and Disadvantage.

With regard to socioeconomic characteristics, 7 individual characteristics were used for the 22 articles and 2 ecological measures for 7 articles (32%), including a specific deprivation index (Index of Relative Socioeconomic Advantage and Disadvantage [IRSAD index]). According to the classification of the individual socioeconomic measures (“complete” or “partial”), only 2 articles measured the socioeconomic position “completely” (9%). For the 20 other articles, 5 did not explore any of the 3 main individual measures (23%).

### Relationship between socioeconomic characteristics, TBI, and costs

Associations among socioeconomic characteristics, TBI incidence and complications, and costs described in papers were summarized. Table III conceptually classifies the articles according to 2 positions: the left column determined that socioeconomic characteristics were related to the occurrence of TBI and its costs. Most of the articles are classified in this position (*n* = 14, 64%). Although 3 articles did not find a significant association between socioeconomic characteristics, injury, and costs (14%) (40, 49, 60), the remaining 11 articles found a significant association between the most disadvantaged categories and increased costs, as well as a higher incidence or complications for the most disadvantaged (50%). Indeed, 6 articles described increased costs for the most disadvantaged (27%) (39, 44, 52, 53, 56, 59), and 7 articles described a more complex incidence or sequelae of injury, such as longer length of stay, development of complications, missed care, or increased mortality (32%) (41, 45, 48, 51–53, 59). The right column determined that socioeconomic characteristics were impacted by the occurrence of a TBI (and its costs). In this sense, the socioeconomic characteristics were modified and were therefore classified as consequences of the TBI and its costs (*n* = 8, 36%). Although Worthington (58) does not identify social role alteration during rehabilitation, 2 other articles point to increased divorce and decreased social interaction as a result of TBI (9%) (42, 47). Three other articles highlight the alteration of income, with a decrease in income and personal resources (43), taking out loans or the separation of property for the family (spouses and parents) to cover the costs associated with TBI (46), and over-indebtedness for TBI victims who have received rehabilitation (14%) (50).

**Table III:** Article viewpoint according to explanatory factors and outcomes of socioeconomic characteristics and costs

Socioeconomic characteristics explain TBI and costs		Socioeconomic characteristics as consequence of TBI and costs	
First author, year of publication	What influences what and about what?	First author, year of publication	What influences what and about what?
Boop, 2016	Costs conditioned by socioeconomic characteristics	Worthington, 2006	TBI affects socioeconomic characteristics and costs of care conditioned by time to care
Spitz, 2016	Costs conditioned by socioeconomic characteristics	Norup, 2020	TBI affects socioeconomic characteristics and costs of care
Dengler, 2020	Social characteristics determine access to care (associated with costs)	Shafi, 2019	TBI affects socioeconomic characteristics and productivity
Tilford, 2005	Occurrence of TBI (and consequences as mortality) depend on the funder (no insurance vs insurance) as well as the production costs	Johnstone, 2003	TBI affects socioeconomic characteristics and requires financial support
Zonfrillo, 2016	Costs conditioned by socioeconomic characteristics	McMordie, 1988	Costs of TBI-related care affect socioeconomic characteristics
Kelly, 2022	Costs conditioned by socioeconomic characteristics	Shigaki, 2009	TBI affects socioeconomic characteristics and requires financial support
Ramey, 2019	Socioeconomic characteristics affect TBI risk and costs	Hart, 2005	Costs of TBI-related care affect socioeconomic characteristics
Piatt, 2012	Funder and socioeconomic characteristics determine the care and prognosis of TBI	Relyea-Chew, 2009	Cost and consequences (disability/need for assistance) of TBI-related care affect socioeconomic characteristics
Klevens, 2017	Occurrence of TBI depends on financial support (and socioeconomic characteristics)		
Schneier, 2006	Funder (Medicaid) affects the cost of care		
Yue, 2020	Socioeconomic characteristics affect costs and access to care		
Reynolds, 2001	Socioeconomic characteristics determine the allocation of funding and access to care		
Graves, 2019	Socioeconomic characteristics affect costs and access to care		
Salik, 2022	Socioeconomic characteristics affect TBI risk and costs		

TBI: traumatic brain injury.

Lastly, 5 articles highlight the impairment of occupation (or employment) with a threefold increase in the risk of job loss (47), an increased risk of absence from work (54), an increase in the number of people who are unemployed for up to 1 year (43), a change in employment for the family (spouses and parents) to cover the costs associated with TBI (46), a return to employment at 2 years for only half of pre-injury TBI and a wage at 2 years for only one-quarter of those who had a wage before the injury (55), and a decrease in productivity after TBI (23%) (42).

## DISCUSSION

The purpose of this scoping review was to provide visibility on the association between socioeconomic characteristics and healthcare costs, in order to enable the evolution of care systems to remain relevant to the populations. Our work has identified very few studies analysing the costs of the care management of TBI victims according to their socioeconomic characteristics. This result highlights the lack of work integrating socioeconomic characteristics into efficiency for TBI management, which is a problem, considering the effect of social characteristics on the care pathway of TBI victims and the long-term consequences. The questions of efficiency and equity still remain unanswered.

Due to the heterogeneity of the articles that motivated the scoping review, all types of economic studies were included in the scoping (30), most of which were studies describing the costs of illness and none of which examined the efficiency of different strategies of care (e.g., cost–benefit analyses, cost–utility, etc.). Thus, the first common methodological element for all economic study designs was to identify the perspectives and cost typologies as recommended and which were missing in most cases (40, 44, 45, 47–49, 53, 55–58, 60). Some articles also sought to adopt a patient perspective to report on the economic consequences of TBI, whereby the out-of-pocket expenses could be a real debt for patients and their families. This may be the case in the USA, where people on low incomes do not have access to non-reimbursed care, so there will be a renunciation of care or even personal bankruptcy (61, 62). Intangible costs were also explored to highlight the impact of TBI in terms of reduced well-being for patients. These consequences can also have an impact on families, with the need to adapt the home, or to change or adapt their working hours in order to be more available to help with care or to cover costs. These results are in line with other authors who highlight the needs of families after the TBI of a relative (63, 64).

Regarding socioeconomic characteristics, we find that the vast majority of indicators used are individual measures but that very few articles explore the 3 main dimensions of socioeconomic position at an individual level (45, 55). We know that these individual variables are poorly recorded in routine care and, in a large proportion of articles, the retrospective design did not improve the quality of the collection and the approximation of socioeconomic position was therefore incomplete for most studies (36). In the absence of individual data, the socioeconomic approximation via ecological indices may be an alternative (32, 65). Currently, there are several validated and available deprivation indicators that allow, from an “easily accessible” variable, approximation of the socioeconomic position. Some US articles have used the household income by zip code as an ecological measure, based on income level (66). And only 1 article was able to use a specific ecological measure of deprivation constructed by crossing deprivation surveys and population census data (IRSAD index) (67).

The cross-analysis of costs and social characteristics allowed us to determine whether socioeconomic characteristics are associated with the occurrence of TBI or whether occurrence of a TBI alters the living conditions. Thus, a conceptual framework could be defined in which socioeconomic characteristics can either be related to the occurrence of a head injury or be impacted as a result of a head injury and its costs. TBI is not randomly distributed in the population and socioeconomic characteristics are involved in the occurrence of TBI. Many of the articles have highlighted that the incidence was higher, the recovery more complex, and the coverage of healthcare higher for the most disadvantaged categories (38–40, 43, 44, 47, 48, 51, 52, 55, 56, 58, 59). These results are in line with those of the authors who point out the higher incidence of TBI in disadvantaged populations (14, 68, 69). Moreover, the cost of care seems to vary according to the socioeconomic characteristics of individuals, but most of them highlight an increased cost of care for disadvantaged groups (39, 41, 44, 52, 53, 56, 59). Lastly, many of the articles highlight the fact that TBI and associated costs can affect living conditions, more particularly alteration of social role, alteration of level of income, and alteration of occupation or employment (42, 43, 46, 47, 50, 54, 55, 58). These results highlight the vulnerability of survivors who undergo long and complex rehabilitation processes with physical and cognitive rehabilitation and social support needs, sometimes for several years after their TBI. These results are consistent with recent results from Arigo and Haggerty (70) in the field of traumatic head injuries. They are also consistent with literature in the field of emergencies or hip fracture, where authors explain that the most disadvantaged populations have a higher cost of care and this relationship is partly due to a different state of health, with higher comorbidity and morbidity in this population (21–23). In a general population, Jayatunga et al. (24) highlight a social gradient, i.e., a gradual increase in costs as deprivation increases.

These elements once again call into question the assessment of severity, which is mainly based on clinical criteria at the time of initial management (use of the Glasgow coma scale). This assessment is currently contested as it is not a good indicator of the complex pathophysiology of TBI and disability (71). Indeed, although clinical assessment remains essential in guiding management and preventing complications, classification on these criteria is insufficient to guide prognosis and the therapies implemented are sub-optimal. Imaging, biomarkers, and pathology should be considered in the diagnostic criteria (72).

Occurrence of TBI appears to alter living conditions in our review. This work therefore suggests the need to carry out a detailed evaluation of the link between socioeconomic characteristics and the occurrence of TBI and to evaluate the costs associated with TBI according to these characteristics. Future work should focus on identifying populations at risk of poor recovery or deterioration following a TBI, and developing care pathways specifically for these groups, which acknowledge and work within the reality of the impact of socioeconomic position on recovery. The aim is then to construct more appropriate, effective, and equitable care programmes.

More generally, healthcare decisions should not be based solely on economic criteria, but should be modulated according to the socioeconomic characteristics of the users in order to integrate equity in these decisions. Efficiency and equity are closely linked. Indeed, if socioeconomic characteristics are not considered, healthcare programmes will only be efficient for part of the population and the care of the other users will require the implementation of additional interventions to compensate for the difference in efficiency between the different socioeconomic groups, and will therefore be more expensive. Some studies have demonstrated the added value in terms of cost-saving of taking these characteristics into account (74, 75).

### Strength and limitations

The strength of this work lies in the detailed analysis of costs and socioeconomic characteristics, which allows these elements to be presented in an optimal and comparable way according to current standards. The scoping methodology has been followed in a systematic and rigorous way that permits the presentation of descriptive data from the selected studies and the analysis of their content. The weaknesses of this work are the lack of exhaustiveness of the databases consulted, even if they were targeted to answer the research question in a relevant way. Although searching by MeSH allows for more targeted results, free-text searching is essential for databases that do not have a thesaurus, but the exhaustiveness of the results remains limited. The language searched was filtered to French and English, and grey literature was not explored. These elements imply that some articles may have been missed. Lastly, evaluation of the quality of the data may be overestimated with the use of a general tool, the counterpart of which would be the lack of specificity to qualitative or economic evaluation of health care programmes’ methods.

### Conclusion

To date, few articles have addressed the issue of socioeconomic characteristics in terms of risk or consequences of TBI and their costs. This lack is all the greater because the evaluation of these dimensions is suboptimal in these articles, which rarely follow the recommendations in relation to economic evaluation of healthcare programmes or the variables and indicators approximating deprivation are not used in a satisfactory manner. This work is a major step in highlighting the lack of evaluation of these dimensions, while their involvement and consequences seem to be important in the pathways of TBI patients. This scoping review could guide future research on the rigorous measurement of these indicators in order to model and design optimal care pathways according to individual characteristics.

## Supplementary Material

MAPPING THE COSTS AND SOCIOECONOMIC CHARACTERISTICS INVOLVED IN TRAUMATIC BRAIN INJURIES: A SCOPING REVIEW
