# Supplementary material for: MAPPING THE COSTS AND SOCIOECONOMIC CHARACTERISTICS INVOLVED IN TRAUMATIC BRAIN INJURIES: A SCOPING REVIEW
Source: J Rehabil Med. 2024 Aug 5;56:18311. doi: 10.2340/jrm.v56.18311 (PMC11318505; doi:10.2340/jrm.v56.18311)
Supplement: MAPPING THE COSTS AND SOCIOECONOMIC CHARACTERISTICS INVOLVED IN TRAUMATIC BRAIN INJURIES: A SCOPING REVIEW [file JRM-56-18311-s1.pdf]

Supplementary material has been published as submitted. It has not been copyedited, or typeset by Journal of Rehabilitation Medicine

Table SI. Search terms

Web of Science<br>((((((TS=(head*)) OR TS=(brain*)) OR TS=(cranium*)) AND TS=(injury*)) OR TS=(trauma*)) AND TS=(socioeconomic*)) AND TS=(cost*)
Pubmed:<br>("economics"[MeSH Terms]) AND (("socioeconomic factors"[MeSH Terms]) AND ("craniocerebral trauma"[MeSH Terms]))
Cochrane Library (via Wiley):<br>MeSH descriptor: [Socioeconomic Factors] explode all trees AND MeSH descriptor: [Economics] explode all trees AND MeSH descriptor: [Craniocerebral Trauma] explode all trees
EMBase:<br>"healthcare cost"/mj AND "head injury"/mj AND "socioeconomics"/mj
List of synonyms searched within thesauri by the Mesh search:<br>(((((((((“craniocerebral trauma”[MH] OR (“head injuries, multiple”[TW] OR “frontal region traumas”[TW] OR “frontal region trauma”[TW] OR “region trauma, frontal”[TW] OR “head injury, multiple”[TW] OR “head injury, superficial”[TW] OR “forehead trauma”[TW] OR “open head injury”[TW] OR “occipital region trauma”[TW] OR “superficial head injuries”[TW] OR “trauma, head”[TW] OR “region trauma, occipital”[TW] OR “head injury”[TW] OR “trauma, occipital region”[TW] OR “occipital trauma”[TW] OR “skull injury, crushing”[TW] OR “superficial head injury”[TW] OR “traumas, craniocerebral”[TW] OR “multiple head injuries”[TW] OR “region trauma, parietal”[TW] OR “crushing skull injury”[TW] OR “parietal region traumas”[TW] OR “injuries, craniocerebral”[TW] OR “injury, open head”[TW] OR “trauma, forehead”[TW] OR “craniocerebral trauma”[TW] OR “head injuries”[TW] OR “trauma, temporal region”[TW] OR “forehead traumas”[TW] OR “traumas, forehead”[TW] OR “head injury, open”[TW] OR “head injury, minor”[TW] OR “trauma, craniocerebral”[TW] OR “injuries, head”[TW] OR “minor head injury”[TW] OR “injury, superficial head”[TW] OR “crushing skull injuries”[TW] OR “traumas, head”[TW] OR “occipital traumas”[TW] OR “trauma, frontal region”[TW] OR “multiple head injury”[TW] OR “region traumas, parietal”[TW] OR “craniocerebral traumas”[TW] OR “trauma, parietal region”[TW] OR “head injuries, superficial”[TW] OR “open head injuries”[TW] OR “minor head injuries”[TW] OR “temporal region traumas”[TW] OR “traumas, frontal region”[TW] OR “head injuries, open”[TW] OR “head traumas”[TW] OR “injury, head”[TW] OR “injuries, multiple head”[TW] OR “skull injuries, crushing”[TW] OR “injuries, superficial head”[TW] OR “occipital region traumas”[TW] OR “injuries, open head”[TW] OR “traumas, parietal region”[TW] OR “temporal region trauma”[TW] OR “traumas, temporal region”[TW] OR “injury, multiple head”[TW] OR “trauma, occipital”[TW] OR “head trauma”[TW] OR “parietal region trauma”[TW] OR “injuries, minor head”[TW] OR “injury, craniocerebral”[TW] OR “injury, minor head”[TW] OR “craniocerebral injuries”[TW] OR “traumas, occipital region”[TW] OR “craniocerebral injury”[TW] OR “region traumas, frontal”[TW] OR “head injuries, minor”[TW] OR “region traumas, occipital”[TW] OR “traumas, occipital”[TW]))))) OR ((“craniocerebral trauma”[MH] OR (“head injuries, multiple”[TW] OR “frontal region traumas”[TW] OR “frontal region trauma”[TW] OR “region trauma, frontal”[TW] OR “head injury, multiple”[TW] OR “head injury, superficial”[TW] OR “forehead trauma”[TW] OR “open head injury”[TW] OR “occipital region trauma”[TW] OR “superficial head injuries”[TW] OR “trauma, head”[TW] OR “region trauma, occipital”[TW] OR “head injury”[TW] OR “trauma, occipital region”[TW] OR “occipital trauma”[TW] OR “skull injury, crushing”[TW] OR “superficial head injury”[TW] OR “traumas, craniocerebral”[TW] OR “multiple head injuries”[TW] OR “region trauma, parietal”[TW] OR “crushing skull injury”[TW] OR “parietal region traumas”[TW] OR “injuries, craniocerebral”[TW] OR “injury, open head”[TW] OR “trauma, forehead”[TW] OR “craniocerebral trauma”[TW] OR “head injuries”[TW] OR “trauma, temporal region”[TW] OR “forehead traumas”[TW] OR “traumas, forehead”[TW] OR “head injury, open”[TW] OR “head injury, minor”[TW] OR “trauma, craniocerebral”[TW] OR “injuries, head”[TW] OR “minor head injury”[TW] OR “injury, superficial head”[TW] OR “crushing skull injuries”[TW] OR “traumas, head”[TW] OR “occipital traumas”[TW] OR “trauma, frontal region”[TW] OR “multiple head injury”[TW] OR “region traumas, parietal”[TW] OR “craniocerebral traumas”[TW] OR “trauma, parietal region”[TW] OR “head injuries, superficial”[TW] OR “open head injuries”[TW] OR “minor head injuries”[TW] OR “temporal region traumas”[TW] OR “traumas, frontal region”[TW] OR “head injuries, open”[TW] OR “head traumas”[TW] OR “injury, head”[TW] OR “injuries, multiple head”[TW] OR “skull injuries, crushing”[TW] OR “injuries, superficial head”[TW] OR “occipital region

traumas"[TW] OR "injuries, open head"[TW] OR "traumas, parietal region"[TW] OR "temporal region trauma"[TW] OR "traumas, temporal region"[TW] OR "injury, multiple head"[TW] OR "trauma, occipital"[TW] OR "head trauma"[TW] OR "parietal region trauma"[TW] OR "injuries, minor head"[TW] OR "injury, craniocerebral"[TW] OR "injury, minor head"[TW] OR "craniocerebral injuries"[TW] OR "traumas, occipital region"[TW] OR "craniocerebral injury"[TW] OR "region traumas, frontal"[TW] OR "head injuries, minor"[TW] OR "region traumas, occipital"[TW] OR "traumas, occipital"[TW]))))) AND ((("social class"[MH] OR ("class population, middle"[TW] OR "social class"[TW] OR "populations, middle class"[TW] OR "classes, social"[TW] OR "class, social"[TW] OR "class populations, middle"[TW] OR "middle class population"[TW] OR "population, middle class"[TW] OR "middle class populations"[TW] OR "social classes"[TW] OR "caste"[TW] OR "castes"[TW]))) OR (((("social class"[MH] OR ("class population, middle"[TW] OR "social class"[TW] OR "populations, middle class"[TW] OR "classes, social"[TW] OR "class, social"[TW] OR "class populations, middle"[TW] OR "middle class population"[TW] OR "population, middle class"[TW] OR "middle class populations"[TW] OR "social classes"[TW] OR "caste"[TW] OR "castes"[TW]))))))) OR ((("Economic Status"[MH] OR ("Economic Status"[TW]))) OR (((("Economic Status"[MH] OR ("Economic Status"[TW]))))))) OR ((("socioeconomic factors"[MH] OR ("factor, socioeconomic"[TW] OR "land tenure"[TW] OR "socioeconomic aspects"[TW] OR "populations, high-income"[TW] OR "living standard"[TW] OR "social inequalities"[TW] OR "factors, socioeconomic"[TW] OR "socioeconomic factors"[TW] OR "high-income populations"[TW] OR "inequalities, social"[TW] OR "high income population"[TW] OR "inequity"[TW] OR "living standards"[TW] OR "standard of living"[TW] OR "high-income population"[TW] OR "population, high-income"[TW] OR "tenure, land"[TW] OR "socioeconomic factor"[TW] OR "inequality, social"[TW]))) OR (((("socioeconomic factors"[MH] OR ("factor, socioeconomic"[TW] OR "land tenure"[TW] OR "socioeconomic aspects"[TW] OR "populations, high-income"[TW] OR "living standard"[TW] OR "social inequalities"[TW] OR "factors, socioeconomic"[TW] OR "socioeconomic factors"[TW] OR "high-income populations"[TW] OR "inequalities, social"[TW] OR "high income population"[TW] OR "inequity"[TW] OR "living standards"[TW] OR "standard of living"[TW] OR "high-income population"[TW] OR "population, high-income"[TW] OR "tenure, land"[TW] OR "socioeconomic factor"[TW] OR "inequality, social"[TW]))))))) AND ((("healthcare costs"[MH] OR ("healthcare costs"[TW] OR "cost, treatment"[TW] OR "healthcare cost"[TW] OR "costs, treatment"[TW] OR "costs, health"[TW] OR "healthcare costs"[TW] OR "cost, medical care"[TW] OR "costs, healthcare"[TW] OR "costs, medical care"[TW] OR "cost, healthcare"[TW] OR "treatment costs"[TW] OR "treatment cost"[TW] OR "healthcare cost"[TW] OR "health cost"[TW] OR "medical care costs"[TW] OR "cost, healthcare"[TW] OR "medical care cost"[TW] OR "healthcare costs"[TW] OR "costs, healthcare"[TW] OR "cost, health"[TW]))) OR (((("healthcare costs"[MH] OR ("healthcare costs"[TW] OR "cost, treatment"[TW] OR "healthcare cost"[TW] OR "costs, treatment"[TW] OR "costs, health"[TW] OR "healthcare costs"[TW] OR "cost, medical care"[TW] OR "costs, healthcare"[TW] OR "costs, medical care"[TW] OR "cost, healthcare"[TW] OR "treatment costs"[TW] OR "treatment cost"[TW] OR "healthcare cost"[TW] OR "health cost"[TW] OR "medical care costs"[TW] OR "cost, healthcare"[TW] OR "medical care cost"[TW] OR "healthcare costs"[TW] OR "costs, healthcare"[TW] OR "cost, health"[TW]))))))

Table SII. Details of variables presented in articles

First author, Year of publication	Study location	Type of publication (design)	Patients' characteristics	Study sample	Objectives	Cost outcomes	Socioeconomic characteristics (other than gender and age)	Quality of the study
Worthington, 2006	UK	QUANT Multi-center prospective cohort study	Adults with acquired brain injury admitted for residential rehabilitation in four residential rehabilitation services operated by the Brain Injury Rehabilitation Trust (independent sector provider)	133	To update a cost-outcome evaluation, based on a new cohort of adults with severe brain injury, in response to an expansion of neurobehavioral rehabilitation services across the UK	Care prior to and after rehabilitation (charged on the basis of current hourly rates of pay, plus on-costs, for nursing and support staff)<br>Rehabilitation costs (based on the daily rate for placement in a rehabilitation center, standardized fee level at time of the study)	Place of residence measured by the hierarchical Accommodation Rating Scale (10 categories: Alone/with partner independently, with parents independently (pre-morbidly), with parents independently (since injury), supported living in own home, supported living in shared house, supported living in parents' house, residential facility, acute medical hospital, psychiatric hospital, behavior disorders unit)<br>Occupational Status (8 categories: Paid employment or education, Vocational training, Supported employment, Independent homemaker, Volunteer, Recreational activity, No productive activity)	Adequate
Boop, 2016	USA	QUANT Retrospective cohort study	All cases of abusive head trauma that were evaluated at Le Bonheur Children's Hospital from 2009 to 2014	213	To characterize the abusive head trauma population in the geographic area served by Le Bonheur Children's Hospital and calculate incidence rates and costs of hospital charges	Hospital charges (professional fees were not included in the hospital charges)<br>Insurance status (3 categories: private, public, or none)	Race (3 categories: White, African-American, or other)<br>State of residence (5 states: Tennessee, Arkansas, Mississippi, Missouri, or Kentucky)	Good

First author, Year of publication	Study location	Type of publication (design)	Patients' characteristics	Study sample	Objectives	Cost outcomes	Socioeconomic characteristics (other than gender and age)	Quality of the study
Spitz, 2016	Australia	QUANT Retrospective cohort study	Patients admitted to a TBI rehabilitation center in the context of a no-fault accident compensation system administered by the Transport Accident Commission (TAC) or Worksafe, between 1987 and 2003	798	To develop competing predictive, multivariate models of costs accrued from the time of the initial accident and over the subsequent 10 years following injury	Hospital (included acute and rehabilitation hospital costs)<br>Medical (related to specialist medical or surgical consultations, as well as medical visits, including pathology, radiology, and psychiatry)<br>Paramedical (including physiotherapy, speech pathology, occupational therapy, psychology, social work, dental costs, vocational services, such as equipment, training, and counselling, assistance in the home, such as gardening, domestic services, and childminding)<br>Long-term care comprised of costs for attendant care, integration aides in schools, special accommodation, special equipment, living expenses and other community support requirements<br>Costs were inflated according to the Australian Bureau of Statistics (increase by 1% above price movements)<br>No discounting was applied to costs	Years of education (2 categories: less than 12 years or more than 12 years)<br>Marital status (2 categories: married/de facto or widowed/divorced/never married/single/separated)<br>Living location (2 categories: metropolitan or country/interstate)<br>Employment (2 categories: employed/studying or not in the labor force/unemployed)	Strong

First author, Year of publication	Study location	Type of publication (design)	Patients' characteristics	Study sample	Objectives	Cost outcomes	Socioeconomic characteristics (other than gender and age)	Quality of the study
Dengler, 2020	USA	QUANT Retrospective cohort study	All consecutive patients with complicated mild traumatic brain injury who were 18 years old or older in a trauma registry collected between 2004 and 2013<br>cmTBI was defined as GCS of 14 to 15 AND the presence of depressed skull fracture, AND/OR a trauma-related intracranial abnormality on the initial computed tomography scan	1447	To determine what the rates of secondary overtriage were in patients with complicated mild traumatic brain injury and to study how they may affect the allocation of healthcare resources	Transportation charges: from medical records, calculated by determining the average cost per mile for both an ambulance and helicopter. The distances between the tertiary care center and the referring hospitals were calculated using Google Maps. The distance considered was a direct distance for flight and shortest road distance for ambulance, multiplied by the average cost per mile (of helicopter flight or ambulance).<br>Hospitalization charges in medical records<br>Total charges in medical records.<br>Insurance status: 3 categories (self-paid, company (worker's compensation, Medicare, or Medicaid) and other)	Race: 4 categories (Asian, Black, Hispanic, White).	Strong

First author, Year of publication	Study location	Type of publication (design)	Patients' characteristics	Study sample	Objectives	Cost outcomes	Socioeconomic characteristics (other than gender and age)	Quality of the study
Norup, 2020	Denmark	QUANT Retrospective case-control study	Patients (TBI) in the Danish National Patient Register and selected by ICD-10 codes between 2008 and 2016<br>Each patient with TBI was matched with five controls based on a propensity score comprising age, gender, years of education, marital status, region, and socioeconomic group. The control population could not have had a TBI but could have had other diseases.<br>The immediate families (cohabiting family members) of both patients and controls were identified in the Danish population register which is based on data from the Danish Civil Registration System.	18,328 TBI<br>89,155 controls<br>25,708 TBI family members<br>135,325 control family members	To investigate if a TBI population had increased i) utilization of healthcare costs for the patient and the closest relatives, ii) risk of job loss for the patient and the closest relatives, and iii) risk of divorce, compared to a matched healthy control group	Healthcare costs: measured in monetary terms, Danish kroner were converted to US dollars at the average exchange rate in 2017<br>Hospital costs were valued by national diagnose related group rates<br>Medical costs were calculated using those for general practitioners and practicing specialists valued at the rates negotiated between the primary healthcare associations and the Danish Regions<br>Prescribed medication valued at the full retail price<br>Job loss: attachment (or not) to the labor market was assessed using the Danish Register for Evaluation of Marginalization database administered by the Ministry of Employment (weekly data availability)	Marital status (5 categories: divorced, married, widow, single, missing value)<br>Annual income (5 categories: $0 to $20,000; $20,000 to $40,000; $40,000 to $60,000; more than $60,000, missing value)<br>National origin (4 categories: Danish, European Union or USA, Rest of the World, missing value)	Strong

First author, Year of publication	Study location	Type of publication (design)	Patients' characteristics	Study sample	Objectives	Cost outcomes	Socioeconomic characteristics (other than gender and age)	Quality of the study
Tilford, 2005	USA	QUANT Retrospective cohort study	Patients aged 0 to 21 years with a diagnosis code indicative of TBI (ICD-9) whose date were available the Nationwide Inpatient Sample from 1988 to 1999	98,023	To examine the incidence, utilization of intracranial pressure monitoring, and outcomes for critically ill children hospitalized with traumatic brain injury	Costs and benefits evaluation: benefits were evaluated in lives lost due to lack of insurance and mortality rates were predicted from a logistic regression model and the estimated incidence of critically ill children with TBI over the study period, assuming a life expectancy of 50 additional years, a value of $100,000 per life year, and a discount rate of 3%<br>Acute care costs were calculated in dollars using the hospital component of the Consumer Price Index to adjust total charges from the NIS database and then applying average cost-to-charge ratios. The total costs over the study period and the average cost per patient were calculated<br>Insurance status (4 categories: public, private, self-pay, other insurance)	Median household income for the patient's zip code of residence (4 categories: low, medium, medium high, high).<br>Race (5 categories: White, Black, Hispanic, Asian/Pacific Islander, other).<br>Hospital characteristics as teaching status and region of the country (3 categories: rural, urban/nonteaching, urban/teaching).	Strong

First author, Year of publication	Study location	Type of publication (design)	Patients' characteristics	Study sample	Objectives	Cost outcomes	Socioeconomic characteristics (other than gender and age)	Quality of the study
Zonfrillo, 2016	USA	QUANT Retrospective cohort study	Patients aged 0 to 18 years with a severe TBI and Spinal Cord Injuries (ICD-9) that represented abbreviated injury scale scores of 4, 5, or 6 in the Pediatric Health Information System, from 2009 to 2012	1061	To examine the association between zip code-level median annual household income and costs of hospitalization among severely injured children	Standardized hospital costs: each service or activity was assigned a standardized cost. These costs were categorized into total, laboratory, imaging, pharmacy, clinical, supply, room, and other.<br>Payer (4 categories: public, commercial/private/employer-based, uninsured, other).	Race (6 categories: White, Black, Hispanic or Latino, Asian, American Indian/Alaskan native, other)<br>Zip code-based median household income: divided into 4 categories based on the US federal poverty level (FPL) for a family of 4. These categories were household income-1, less than 1.5 times the FPL ($34,575 or less); household income-2, 1.5-2 times the FPL ($34,576-$46,100); household income-3, 2-3 times the FPL ($46,101-$69,150), and household income-4, greater than 3 times the FPL ($69,151 or more)	Strong
Shafi, 2019	Australia	QUANT Retrospective cohort study	Workers' compensation claimants in Australia who had sustained a work-related mild TBI in the Compensation Research Database and severity was determined using hospital admissions, from 2004 to 2012	3129	To characterize the sample of workers who had sustained a work-related mild TBI and to assess the influence of assault, as a mechanism of injury, on time away from work	Time away from work post-injury: workers who took time off work within the first 90 days post-affliction (binary variable)	Socioeconomic position coded into 10 deciles, from 1 (disadvantaged) to 10 (advantaged) based on the Index of Relative Socioeconomic Advantage and Disadvantage<br>Employment type (3 categories: full-time (working ≥35hours/week), part-time (<35hours/week), and other (e.g., apprentice, work placement student, group trainee, volunteer, etc.))<br>Occupational group (4 categories: professionals, community and personal service workers, service-related occupations (managers, technicians and trades workers, clerical and administrative workers, sales workers, etc.), and goods-related occupations (machinery operators, drivers, and laborers))<br>binary variable "absence from work of at least 2 weeks in the 90 days following the TBI"	Strong

First author, Year of publication	Study location	Type of publication (design)	Patients' characteristics	Study sample	Objectives	Cost outcomes	Socioeconomic characteristics (other than gender and age)	Quality of the study
Kelly, 2022	USA	QUANT Retrospective cohort study	Patients aged 0 to 19 years with ICD-9 codes for TBI in the Nationwide Inpatient Sample database from 2012 to 2015	19,848	To evaluate socioeconomic and health disparities among children hospitalized after a TBI	Cost of the hospital stay calculated from the reported total charge by using Healthcare Cost and Utilization Project cost-to-charge ratios	Race (6 categories: White, Black, Hispanic, Asian or Pacific Islander, Native American, or other). Median income of the zip code of residence (stratified into income quartiles, with 1 representing the lowest and 4 representing the highest income quartile) Geographic region (8 categories: Mountain, Pacific, West North Central, West South Central, East North Central, East South Central, South Atlantic, Middle Atlantic, and New England).	Strong
Johnstone, 2003	USA	QUANT Prospective longitudinal study	Patients with new TBI from one national Traumatic Brain Injury Model Systems center and had both critical care and rehabilitation	35	To characterize financial and vocational outcomes among persons with a TBI in terms of employment status, earned and private income, and public assistance received at the time of injury and at one year after the injury	Average government financial assistance per month (including Supplemental Security Income, Social Security Disability Income, Temporary Assistance for Needy Families/Aid to Families with Dependent Children, general welfare, and/or other public assistance)	Employment status (4 categories: employed, unemployed, student, and retired/homemaker/other) Average earned and private income per month (including earned income, family support, other private income, and total private income)	Good

First author, Year of publication	Study location	Type of publication (design)	Patients' characteristics	Study sample	Objectives	Cost outcomes	Socioeconomic characteristics (other than gender and age)	Quality of the study
McMordie, 1988	USA	QUANT Cross-sectional survey	People with a head injury and their family members who were members of the Iowa Head Injury Association	28 people with a head injury, 100 parents, 50 spouses, and 11 siblings	To explore various parameters of the financial costs of head injury for the family	Different costs were requested from and reported (average costs) by parents and spouses as of the injury or on a yearly basis: doctors and hospital bills, medications and others medical expenses, legal expenses, structural modifications in the home, specialized therapy, other	Yearly family income (average costs).<br>Other financial implications were reported (binary variable: unemployed family member had to go to work, had to borrow money, had to lose possessions, and/or had to declare bankruptcy).	Good
Shigaki, 2009	USA	QUANT Prospective longitudinal study	Patients with a new TBI, enrolled in one US national Traumatic Brain Injury Model Systems center, who received acute inpatient and rehabilitation services and had available financial and employment data	49	To examine the long-term financial and vocational outcomes for TBI at two years' post-injury	Public assistance: N reporting financial data and total costs per month	Race (3 categories: White, Black, Hispanic)<br>Marital status (5 categories: single, married, divorced, separated, widowed)<br>Education level (5 categories: college degree, completed some college, completed trade school, high school degree, <high school degree)<br>Residence (2 categories: urban, rural)<br>Employment status (4 categories: employed, unemployed, retired/homemaker/other, or student)<br>Earned income (mean, USD)	Good

First author, Year of publication	Study location	Type of publication (design)	Patients' characteristics	Study sample	Objectives	Cost outcomes	Socioeconomic characteristics (other than gender and age)	Quality of the study
Ramey, 2019	USA	QUANT Retrospective case review	Patients treated at Banner University of Arizona Medical Center Tucson from 2012 to 2017, identified as having jumped or fallen from the border wall, who sustained cranial or spinal trauma	64	To examine patterns of neurotrauma and associated healthcare utilization in patients treated as a result of unauthorized border crossings by jumping over the US-Mexico border wall	Physician total charges and those that were reimbursed<br>Inpatient hospital charges and those that were reimbursed	Ethnicity (3 categories: Latin American, Eastern Indian, Asian)	Adequate

First author, Year of publication	Study location	Type of publication (design)	Patients' characteristics	Study sample	Objectives	Cost outcomes	Socioeconomic characteristics (other than gender and age)	Quality of the study
Piatt, 2012	USA	QUANT Retrospective cohort study	Children with a severe TBI identified on the basis of ICD-9 codes and who have a principal or other diagnosis on the Kids' Inpatient Database from 1997 to 2009	14,932	To document the impact of changes during the end of the last century and the beginning of the current one (automotive safety engineering, car seats, bicycle helmets, development of tertiary children's medical centers, protocol driven management in the healthcare facilities, etc.) to the practice of neurosurgery on children with a severe TBI	Hospital charges adjusted for inflation in comparison with charges in 2009 by using the Medical Care Services component of the Consumer Price Index<br>Primary expected payer (4 categories: Medicaid, commercial including healthcare organization, self-pay, other (Medicare, no charge, and other))	Race (4 categories: White, Black, Hispanic, other (Asian or Pacific Islander, Native American, or other))<br>Median household income of the patient's home zip code (ordinal variable in 4 grades: from lowest to highest)	Good

First author, Year of publication	Study location	Type of publication (design)	Patients' characteristics	Study sample	Objectives	Cost outcomes	Socioeconomic characteristics (other than gender and age)	Quality of the study
Hart, 2005	USA	QUANT Prospective longitudinal study with retrospective self-assessment	Patients recruited from consecutive admissions to a dedicated TBI rehabilitation program at an urban rehabilitation hospital with one-year follow-up, with moderate to severe TBI	94	To investigate the contribution of pre-injury differences and potential biases in outcome measurement in explaining outcome differences between white and Black persons with moderate and severe TBI	Productivity (subscale of the Community Integration Questionnaire) with household income dimension and hours worked per week dimension	Demographic and injury variables analyzed by race (Whites, Black)<br>Education (years)<br>Marital status or living situation (2 categories: Married/cohabitation, living alone)	Good
Klevens, 2017	USA	QUANT Retrospective case-control study	Children under the age of two identified in the State Inpatient Databases (available for 14 states with EITCs and 13 states without EITCs) from 1995 to 2013. Abusive head trauma was identified using the International Classification of Diseases and External Cause of Injury diagnosis codes.	380	To examine whether states' earned income tax credits (EITC) are associated with state rates of hospital admissions for abusive head trauma among children under the age of two among states with and without a state EITC	EITC (3 categories: 1)- in states without the EITC, 2)- in states with a nonrefundable EITC (i.e., tax filer only gets credit for any tax owed), and 3)- in states with a refundable EITC (i.e., tax filer gets money even if taxes are not owed))	In the adjusted model, percentage of non-Latinx white population, percentage of the population over the age of 25 who graduated from high school, the states' yearly unemployment rate, and child poverty rates were added as covariates	Good

First author, Year of publication	Study location	Type of publication (design)	Patients' characteristics	Study sample	Objectives	Cost outcomes	Socioeconomic characteristics (other than gender and age)	Quality of the study
Schneier, 2006	USA	QUANT Retrospective cohort study	Children aged 17 and under with a diagnosis of traumatic brain injury identified using the ICD-9, with data from the Kids' Inpatient Database, from January 1, 2000, to December 31, 2000	25,783	To examine the influence of sociodemographic characteristics and healthcare system factors on the utilization of hospital resources by US children aged 17 and under with a diagnosis of traumatic brain injury	Total charges reported on the Kids' Inpatient Database (does not include professional fees and noncovered charge). Values are rounded to the nearest dollar, to set 0 charges to "missing", and to set excessively high (more than $1 million) or excessively low (less than $25) charges to "inconsistent"<br>Primary expected payer (5 categories: Medicaid, private, self-pay, other (Medicare, no charge, and other), not specified)	Race (4 categories: White, Black, other, not specified)	Strong

First author, Year of publication	Study location	Type of publication (design)	Patients' characteristics	Study sample	Objectives	Cost outcomes	Socioeconomic characteristics (other than gender and age)	Quality of the study
Relyea-Chew, 2009	USA	QUANT Cross-sectional survey	Patients aged 20 years or older, residing in western Washington State, were selected from the Seattle, WA, Harborview Medical Center trauma registry, hospitalized between 1996, and 2002, with TBI or SCI identified using ICD-9. Data were linked to the Western District of Washington state bankruptcy court database who filed for bankruptcy. A random selection of petitioners who had not been hospitalized with TBI or SCI and filing for bankruptcy in the same district during the same time period was selected.	186 (93 TBI or SCI who filed for bankruptcy + 93 controls)	To estimate the prevalence of bankruptcy with substantial medical debt, comparing bankruptcy petitioners with TBI and SCI with a cohort of randomly selected bankruptcy petitioners	Medical care sponsor (Medicaid, commercial, Medicare, workers' compensation, other/missing), Bankruptcy (2 categories: liquidation and adjustment of debts with regular income) Median secured debts (e.g., a mortgage) Median unsecured debts (priority debt (e.g., taxes or alimony), nonpriority debts (e.g., credit card), and debts owed to medical or dental service providers) Debtor median monthly medical and dental expenses	Race (2 categories: white or non-white). Mean of household income by zip code Percentage of divorced or separated	Strong
Yue, 2020	International	QUAL Literature review	keywords "rural" and "concussion" or "mild traumatic brain injury" from 1991 to 2019	18 articles	To provide a comprehensive review of the current evidence on rural mTBI/concussion epidemiology, risk factors, management, and prevention efforts in rural settings	Insurance status and healthcare costs were outcome measures in different selected articles	Area of residence and employment status were outcomes measures in different selected articles	Good

First author, Year of publication	Study location	Type of publication (design)	Patients' characteristics	Study sample	Objectives	Cost outcomes	Socioeconomic characteristics (other than gender and age)	Quality of the study
Reynolds, 2001	USA	QUAL Exploratory survey	State administrators to each state-led agency listed in the National Association of State Head Injury Administrators and trauma center social workers to each of the 17 TBI model systems	42 state administrators and 12 trauma center social workers	To identify and describe the barriers faced by patients with a new TBI and evaluate to what extent state Medicaid programs fund post-acute rehabilitation services	No direct measures but the survey is about an uninsured, indigent TBI patient who needs post-acute care, potentially eligible for Medicaid.<br>Participants were asked during interviews: if their state has a home and community-based waiver for these patients, if their state has a Medicaid waiver program that would cover post-acute care for these patients, if alternative programs/funding streams are available	No direct measures but the survey is about an uninsured, indigent TBI patient who needs post-acute care, potentially eligible for Medicaid. Participants were asked during interviews: if the state has a presumptive eligibility policy for recently injured individuals with TBI, what happens to an indigent TBI patient with no coverage, if there are effects of Medicaid delay on care received	Good
Graves, 2019	USA	QUANT Retrospective cohort study	Children (younger than 18 years of age) with an outpatient claim associated with a TBI diagnosed with ICD-9 codes and using the ICD Programs for Injury Categorization to categorize severity, with data from the Commercial Claims and Encounters database, from 2007 to 2011	387,846	To compare healthcare costs and service utilization associated with mild TBI in rural and urban commercially insured children	Total healthcare costs (sum of all outpatient services)<br>Costs for physical therapy or occupational therapy (sum of all payments, based on Current Procedural Terminology codes, revenue and provider type codes)<br>Speech therapy (sum of all payments, based on Current Procedural Terminology codes, revenue and provider type codes)<br>Psychiatry/psychology encounters (sum of all payments, based on Current Procedural Terminology codes, revenue and provider type codes)	Rural residence (2 categories: urban area or rural area<br>Geographic region (4 categories: northeast, north central, south, or west)	Strong

EITC: Earned Income Tax Credits; ICD: International Classification of Diseases; QUANT: QUANTitative design; QUAL: QUALitative design; TBI: Traumatic Brain Injury; SCI: Spinal Cord Injury.
